# The Emerging Vision of the *JACMP* – Part 3

**DOI:** 10.1120/jacmp.v14i4.4551

**Published:** 2013-07-08

The future of the *JACMP* begins with defining our vision of what a 21^st^ century open‐access journal should look like. Yes, money is very tight. Still, the consensus is that the *JACMP* is serving the needs of the community and we believe the community will find a way to support it. So, given our boundless optimism that the money issues will eventually take care of themselves, let us flesh out some details of what the *JACMP* would look like if we could have everything we wanted. We will discuss the publication model, financial issues, continuing education activities, and management concerns. We have teams of Section Editors who have volunteered to look after many of the goals discussed below.

## PUBLICATION MODEL

A primary goal is to identify a low‐cost application (app) that would allow users of iPhone and Android technologies to view and use the content of the *JACMP*. Thus far, the quotes we have seen for such apps are pretty costly. Still, we have not given up hope that we will find a solution for our users who prefer these platforms to conduct their research. A good application should provide a Title Cover, which could depict any good graph or figure from one of our last year's best paper award winners. Each paper in that issue could be like a bookmark in a pdf format or an electronic book, providing easy access to the user. Between the papers, we can add advertisements. The back page will also have advertisements. This could open a way to recognize last year's awardees’ works, and will open a new stream of advertisement revenue. Another area of discussion is the “impact factor” of the *JACMP*. It could be argued that the impact factor is of less importance for a clinical journal. However, the clinical faculties at major universities are coming under more pressure to prove their scholarship and for those who publish in the *JACMP*, a higher impact factor would not hurt. Some techniques used by journals to raise their impact factor are perhaps a little nefarious:

- Publish higher quality articles at the expense of publishing many fewer submissions. This has the added benefit of lowering costs.
- Solicit review articles that have high citation impact.
- Require authors to cite 3–4 *JACMP* articles with each submission. Some academic journals have such rules and the effect on the impact factor is dramatic.
- Require Section Editors to cite 3–4 *JACMP* articles in every submission to other academic journals.
- Devote, on occasion, an entire issue to a special hot topic(s).

As you see, some of these techniques are ethically a little questionable. Nevertheless, these are actually pretty common publication practices for journals concerned with their impact factor.

## FINANCES

Since its inception, the *JACMP* has provided approximately 800 open‐access academic articles to the medical physics community without cost of any kind. It is common for the author cost to be in the thousands of dollars to publish open‐access in some other journals. I discussed my ideas on finances within the Editorial in the previous issue. Still, I want to remind all of you that the money to pay for the *JACMP* needs to come from somewhere. If the money does not come from the author, then where should it come from_ The business model of the *JACMP* is under active discussion this year.

## CONTINUING EDUCATION

It may be possible to arrange to award continuing education (CE) credits for writing an accepted article or reviewing an article. We should figure out the mechanisms for awarding Self‐Assessment Modules (SAMS) credits for such activities. So much of the work we all do for the *JACMP* receives very little credit respecting academic accomplishment. We need to do what we can to remedy that deficiency.

## *JACMP* MANAGEMENT

Some ideas we are considering include our own version of Point–Counterpoint specific to clinical and professional issues, a Book Review Section (we would need a Book Review Editor), and ways to expand the Administrative/Professional Section of the *JACMP*.

As you see we have our work cut out for us. I will provide a report on our progress in these areas from time to time.

Michael D. Mills Editor‐in‐Chief May 27, 2013

